# Pathogenesis of ectodermal dysplasia

**DOI:** 10.1186/1746-160X-8-S1-I8

**Published:** 2012-05-25

**Authors:** Irma Thesleff

**Affiliations:** 1 Institute of Biotechnology, University of Helsinki, Finland

## 

Ectodermal dysplasias (ED) are characterized by impaired development of organs forming from the embryonic surface ectoderm. Thus, in ED organs like teeth, hair, nails and exocrine glands are hypoplastic or totally missing. The pathogenesis of the defects is starting to be understood thanks to the identification of the responsible gene mutations, and to the advances in developmental biology. Rapid progress particularly in genetics and in the generation of genetically modified animals such as transgenic mice has allowed the exploration of events leading to ectodermal organ defects at the cellular and molecular levels. Mouse models have been produced for many different ED syndromes. Most work so far has focused on hypohidrotic ED which is caused by mutations affecting the ectodysplasin (Eda) signalling pathway. Analyses of mouse mutants in which Eda signalling is either blocked (like in most patients with hypohidrotic ED) or overactivated have indicated that Eda signalling is a key regulator of ectodermal placodes. The placodes initiate the formation of ectodermal organs, their positions determine the location of these organs, and their size is associated with organ size. Eda signalling also affects later stages of development, influencing for instance the shape of teeth and branching of salivary and mammary glands. Recent data indicate that Eda signalling is intimately linked to many other signalling pathways, e.g. Wnt, BMP, and FGF pathways, which regulate cell communication together with Eda. The detailed understanding of the functions of these molecular networks can be expected to lead to new possibilities to prevent and treat ED.

